# Impact of initial coagulation and fibrinolytic markers on mortality in patients with severe blunt trauma: a multicentre retrospective observational study

**DOI:** 10.1186/s13049-019-0606-6

**Published:** 2019-02-28

**Authors:** Kenta Ishii, Takahiro Kinoshita, Kazutaka Kiridume, Atsushi Watanabe, Kazuma Yamakawa, Shota Nakao, Satoshi Fujimi, Tetsuya Matsuoka

**Affiliations:** 1 Department of Trauma and Critical Care, Rinku General Medical Centre, Senshu Trauma and Critical Care Centre, 2-23 Rinku Orai-kita, Izumisano, Osaka, 598-8577 Japan; 2 Division of Trauma and Surgical Critical Care, Osaka General Medical Centre, 3-1-56 Bandai-Higashi, Sumiyoshi-ku, Osaka, 558-8558 Japan

**Keywords:** Coagulopathy, D-dimer, Fibrinogen, Classification and regression tree, Logistic regression, Receiver operating characteristic curve, Prediction model

## Abstract

**Background:**

Acute coagulopathy is a well-known predictor of poor outcomes in patients with severe trauma. However, using coagulation and fibrinolytic markers, how one can best predict mortality to find out potential candidates for treatment of coagulopathy remains unclear. This study aimed to determine preferential markers and their optimal cut-off values for mortality prediction.

**Methods:**

We conducted a retrospective observational study of patients with severe blunt trauma (injury severity score ≥ 16) transferred directly from the scene to emergency departments at two trauma centres in Japan from January 2013 to December 2015. We investigated the impact and optimal cut-off values of initial coagulation (platelet counts, fibrinogen and prothrombin time-international normalised ratio) and a fibrinolytic marker (D-dimer) on 28-day mortality via classification and regression tree (CART) analysis. Multivariate logistic regression analysis confirmed the importance of these markers. Receiver operating characteristic curve analyses were used to examine the prediction accuracy for mortality.

**Results:**

Totally 666 patients with severe blunt trauma were analysed. CART analysis revealed that the initial discriminator was fibrinogen (cut-off, 130 mg/dL) and the second discriminator was D-dimer (cut-off, 110 μg/mL in the lower fibrinogen subgroup; 118 μg/mL in the higher fibrinogen subgroup). The 28-day mortality was 90.0% (lower fibrinogen, higher D-dimer), 27.8% (lower fibrinogen, lower D-dimer), 27.7% (higher fibrinogen, higher D-dimer) and 3.4% (higher fibrinogen, lower D-dimer). Multivariate logistic regression demonstrated that fibrinogen levels < 130 mg/dL (adjusted odds ratio [aOR], 9.55; 95% confidence interval [CI], 4.50–22.60) and D-dimer ≥110 μg/mL (aOR, 5.89; 95% CI, 2.78–12.70) were independently associated with 28-day mortality after adjusting for probability of survival by the trauma and injury severity score (TRISS Ps). Compared with the TRISS Ps alone (0.900; 95% CI, 0.870–0.931), TRISS Ps with fibrinogen and D-dimer yielded a significantly higher area under the curve (0.942; 95% CI, 0.920–0.964; *p* < 0.001).

**Conclusions:**

Fibrinogen and D-dimer were the principal markers for stratification of mortality in patients with severe blunt trauma. These markers could function as therapeutic targets because they were significant predictors of mortality, independent from severity of injury.

## Background

Haemorrhage plays a critical role in deaths caused by trauma, accounting for approximately 30–40% of trauma-related deaths [1]. Coagulopathy occurring in early phases of trauma leads to systemic haemorrhage that cannot be controlled using surgical procedures; patients who present with coagulopathy following trauma have an approximately four-fold higher mortality [2–4]. These patients are considered to require ‘damage control strategy’ including damage control surgery, restrictive fluid administration, massive transfusion and use of antifibrinolytic drugs. Therefore, detection of coagulopathy in patients with trauma is crucial for providing adequate management.

Prolonged prothrombin time has been defined as coagulopathy that increases risk of death in patients with severe trauma [2–5]. Decreased platelet counts and fibrinogen levels are also considered important signals because those represent coagulopathy that is potentially amendable with blood transfusions, including platelet concentrate (PC), fresh frozen plasma (FFP), fibrinogen concentrate and cryoprecipitate [4, 6–10]. Additionally, recent studies have reported that elevated D-dimer, which suggests the existence of hyperfibrinolysis, is a significant predictor of poor outcomes [11–13]. Other than those standard laboratory-based coagulation tests, viscoelastic methods are reportedly effective for rapid assessment of coagulopathy [14, 15]; however, several systematic reviews indicate limited evidence to support their utility [16, 17]. Therefore, the European guidelines regarding coagulopathy after trauma still recommend early and repeated monitoring of standard laboratory-based coagulation tests as well as parameters of viscoelastic testing [18].

From these perspectives, traditional coagulation and fibrinolytic markers play important roles in the management of patients with trauma. However, which of these markers best contributes to detecting coagulopathy and stratification of trauma patients at risk for mortality has been poorly investigated. Moreover, huge variations in the cut-off values have been reported to predict mortality of these markers in previous studies [2–5, 9–12]. Thus, how can one best stratify patients with trauma and predict their mortality using coagulation and fibrinolytic markers remains to be established.

The purpose of this study was to investigate the impact of initial coagulation and fibrinolytic markers on mortality in patients with severe trauma. Specifically, we identified preferential markers profitable for prediction of mortality and determined their optimal cut-off values. We hypothesised that combination of coagulation markers (platelet counts, prothrombin time or fibrinogen) and a fibrinolytic marker (D-dimer) would be profitable for predicting mortality.

## Methods

### Study design and patient population

**Fig. 1:**
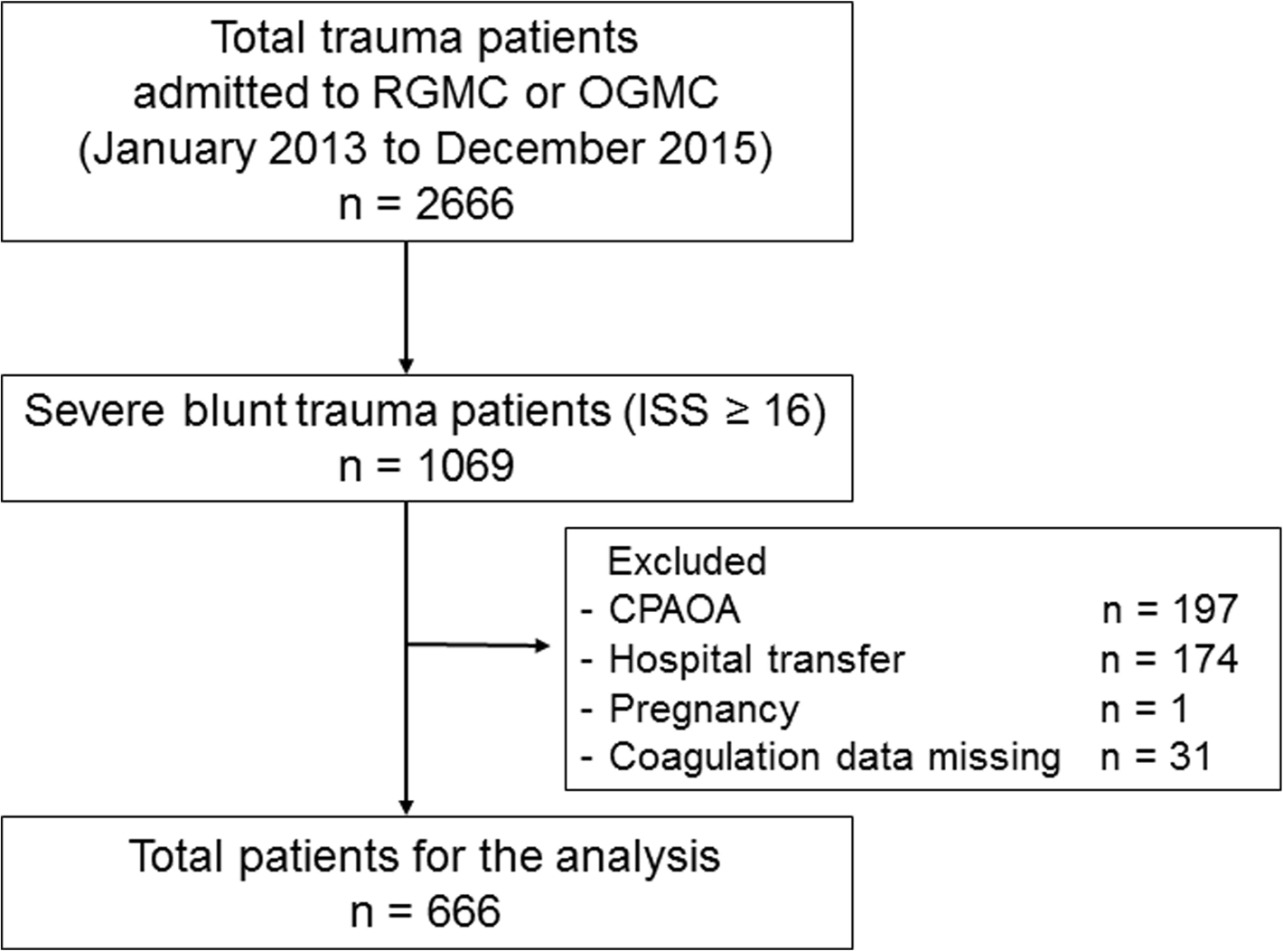
Flow chart depicting the criteria for inclusion of patients in the study. *CPAOA* cardiopulmonary arrest on arrival, ISS injury severity score, *OGMC* Osaka General Medical Centre, *RGMC* Rinku General Medical Centre

In this multicentre retrospective cohort study, we reviewed consecutive severe (injury severity score [ISS] ≥ 16) blunt trauma patients who were admitted to the emergency department (ED) at two trauma centres in Osaka, Japan (Rinku General Medical Centre or Osaka General Medical Centre) between January 2013 and December 2015. We excluded patients who experienced cardiopulmonary arrest on ED arrival, pregnant patients and patients transferred from other hospitals or whose coagulation or fibrinolytic markers were not examined at the ED (Fig. 1).

This study followed the principles of the Declaration of Helsinki and was approved by the institutional ethical review board of Rinku General Medical Centre and Osaka General Medical Centre (#28–39 and #29–S0404, respectively). The boards waived the need for patient consent because of the anonymous and observational nature of this study.

### Data collection

Emergency department variables (systolic blood pressure, heart rate, respiratory rate, Glasgow coma scale and body temperature) were recorded as the initial set of vital signs. We routinely collected blood samples immediately after arrival at the ED before starting infusion and transfusion to examine haemoglobin level, lactate level, base deficit and blood tests regarding coagulation and fibrinolysis including platelet counts, plasma fibrinogen, prothrombin time-international normalised ratio (PT-INR) and D-dimer.

The plasma fibrinogen concentrations were analysed using the modified Clauss method [19]; the same kit (Thrombocheck Fib (L); Sysmex Corporation, Kobe, Japan) was used in the central laboratory of both hospitals. The prothrombin time (Thrombocheck PT; Sysmex, and Tromborel S; Sysmex) and D-dimer (Nanopia D-dimer; Sekisuimedical, Tokyo, Japan, and LIASAUTO D-dimer NEO; Sysmex) were measured using different kits at both hospitals.

The abbreviated injury scale (AIS) of each body region was recorded, and ISS was determined based on the AIS scores. We calculated revised trauma score (RTS) and probability of survival by the trauma and injury severity score (TRISS Ps), which comprised age, ISS and RTS (coefficients: b0, − 1.2470; b1, 0.9544; b2, − 0.0768; b3, − 1.9052) [20].

### Outcome measures

The objective variable in this study was in-hospital, all-cause mortality within 28 days of the injury, including death in the ED. Injury locations, blood transfusion amount (packed red blood cells, FFP and PC), use of antifibrinolytic drugs, mortality within the first 24 h from admission and cause of death were also evaluated. At the time of this study, fibrinogen concentrate and cryoprecipitate were not available in both hospitals. We used tranexamic acid (TXA) as an antifibrinolytic drug when existence of hyperfibrinolysis was clinically suspected. A massive transfusion was defined as a transfusion of ≥10 units of packed red blood cells within the first 24 h. The causes of death were classified into the following groups: exsanguination, traumatic brain injury (TBI), sepsis or multiple organ dysfunction syndrome (MODS) and others. Isolated TBI was defined as no injuries with an AIS score ≥ 3, except for the head injury, and multiple trauma was defined as multiple injuries with an AIS score ≥ 3 in two or more regions.

### Statistical analyses

Continuous variables were expressed as median and interquartile ranges (IQR). Wilcoxon rank sum tests were used for intergroup comparison, because the data were not normally distributed. Categorical variables were expressed as numbers and percentages, and Pearson’s χ^2^ test was used for intergroup comparison, unless the expected scores in any of the cells were ≤ 5; Fisher’s exact test was used in such a situation.

The predictive performance of each coagulation or fibrinolytic marker for 28-day mortality was evaluated using area under the curve (AUC) of the receiver operating characteristic (ROC) curve. The AUCs of the coagulation and fibrinolytic markers were compared with those of base deficit.

We conducted classification and regression tree (CART) analysis to identify preferential markers and their optimal cut-off values for the prediction of 28-day mortality. CART is a nonparametric decision tree methodology that can partition populations into meaningful subsets whose members share similar characteristics [21]. In the CART analysis of this study, platelet counts, fibrinogen, PT-INR and D-dimer were injected as explanatory variables. We set the splitting criterion used in building the decision tree as Gini impurity; minimum improvement as 0.001, which indicates modest differences between the two nodes [21]; minimum cases of parent node as 40; and those of child node as 20 because we considered less than 20 cases of nodes to be too small to have significant meanings. A 20-fold cross-validation was performed to estimate the misclassification cost of the decision tree [21–23]. The characteristics of subgroups generated by the CART analysis were also evaluated.

Finally, we performed a multivariate logistic regression analysis to evaluate the impact of the markers identified through the CART analysis with adjustment for TRISS Ps as an important confounder. Selected markers were treated as binominal variables based on the cut-off values of CART analysis. Specifically, we built a logistic regression model including TRISS Ps and the selected markers, and their odds ratios (ORs) with 95% confidence intervals (CIs) were estimated. The incremental value of adding the selected markers to the TRISS Ps was evaluated by comparing the AUCs of the ROC curves, continuous net reclassification improvement (NRI), and integrated discrimination improvement (IDI) [24]. The *p* values for the comparison of two ROC curves and 95% CIs of the ORs in the logistic regression analysis were calculated using the bootstrap method, which was repeated 2000 times.

All statistical analyses were performed using the IBM SPSS Statistics version 24.0 for Windows (SPSS Inc., Chicago, IL, USA) and R software packages version 3.2.5 for Windows (R Foundation for Statistical Computing, Vienna, Austria). A two-tailed *p* value of < 0.05 was considered statistically significant.

## Results

### Patient characteristics

Among the 2666 trauma patients admitted to the two hospitals during the study period, 1069 severe (ISS ≥ 16) blunt trauma patients were identified. Of these, 403 patients were excluded, and the remaining 666 were included and analysed (Fig. 1). Of the 666 analysed patients, 69 (10.4%) died within 28 days of the injuries. Table 1 summarizes the study population characteristics, and compares the characteristics between survivors and non-survivors. Approximately 20% of all enrolled patients indicated systemic circulatory collapse on ED arrival. All initial laboratory variables were significantly worse in the non-survivors than in the survivors. In contrast, frequency of preinjury anticoagulant/platelet medication was not significantly different between survivors and non-survivors. The number of patients who received interventions is shown in Table 2. Non-survivors received TXA and underwent massive transfusion and emergency surgical procedures more frequently than survivors.

**Table 1:** Baseline characteristics of patients included in the study

	Overall *n* = 666	Survivors *n* = 597	Non-survivors *n* = 69	*P* value
Age, years	52 (31–70)	51 (31–69)	63 (35–78)	0.01
Male sex, *n* (%)	459 (68.9)	416 (69.7)	43 (62.3)	0.22
Anticoagulant/platelet agents, *n* (%)	46 (6.9)	44 (7.4)	2 (2.9)	0.21
Vital signs on ED arrival				
Systolic blood pressure, mmHg	132 (115–153)	132 (116–153)	132 (99–155)	0.40
Heart rate, beats/min	89 (77–105)	89 (77–103)	99 (74–120)	0.07
Shock index ≥1.0, *n* (%)	117 (17.6)	96 (16.1)	21 (30.4)	0.003
Respiratory rate, breaths/min	23 (18–28)	23 (18–27)	21 (16–30)	0.22
Glasgow coma scale	13 (7–15)	15 (9–15)	3 (3–6)	< 0.001
Body temperature, °C	36.4 (36.0–36.7)	36.4 (36.0–36.8)	36.1 (35.2–36.5)	0.002
Initial laboratory variables				
Haemoglobin, g/dL	13.1 (11.6–14.4)	13.3 (11.9–14.4)	11.9 (10.2–13.1)	< 0.001
Lactate, mmol/L	2.4 (1.7–3.8)	2.4 (1.6–3.6)	3.5 (2.3–7.4)	< 0.001
Base deficit, mmol/L	1.2 (−0.4–3.5)	1.1 (−0.5–3.2)	4.4 (0.6–8.7)	< 0.001
Coagulation/fibrinolytic markers				
Platelet count, /μL	216 (169–260)	221 (178–263)	160 (126–205)	< 0.001
PT-INR	1.1 (1.0–1.2)	1.1 (1.0–1.2)	1.4 (1.0–2.0)	< 0.001
Fibrinogen, mg/dL	220 (177–288)	227 (187–284)	122 (71–184)	< 0.001
D-dimer, μg/mL	24.5 (8.8–62.4)	20.6 (7.4–52.2)	141 (59.8–283)	< 0.001
Injury location (AIS ≥ 3)				
Head, *n* (%)	450 (67.6)	390 (65.3)	60 (87.0)	< 0.001
Face, *n* (%)	13 (2.0)	13 (2.2)	0 (0.0)	0.38
Chest, *n* (%)	309 (46.4)	272 (45.6)	37 (53.6)	0.25
Abdomen, *n* (%)	105 (15.8)	87 (14.6)	18 (26.1)	0.02
Extremity/Pelvis, *n* (%)	184 (27.6)	165 (27.6)	19 (27.5)	1.00
Isolated TBI, *n* (%)	227 (34.1)	201 (33.7)	26 (37.7)	0.51
Multiple trauma, *n* (%)	324 (48.6)	283 (47.4)	41 (59.4)	0.07
Injury Severity Score	26 (19–34)	25 (17–30)	38 (26–50)	< 0.001
Revised Trauma Score	7.55 (5.97–7.84)	7.84 (6.38–7.84)	4.09 (3.80–5.82)	< 0.001
TRISS Ps	0.92 (0.66–0.98)	0.94 (0.80–0.98)	0.24 (0.08–0.57)	< 0.001

Data are expressed as medians (25–75 percentiles) or numbers (%). Non-survivors are defined as patients who died within 28 days of injury. The shock index is calculated with heart rate/systolic blood pressure. Isolated TBI is defined as no injuries with an AIS score ≥ 3, except for the head injury. Multiple trauma is defined as multiple injuries with an AIS score ≥ 3 in two or more regions. *AIS* Abbreviated injury scale, *ED* Emergency department, *PT-INR* Prothrombin time-international normalised ratio, *TBI* Traumatic brain injury, *TRISS Ps* probability of survival by trauma and injury severity score

**Table 2:** Interventions

	Overall *n* = 666	Survivors *n* = 597	Non-survivors *n* = 69	*P* Value
Blood transfusions within 24 h				
PRBCs ≥10 units, *n* (%)	105 (15.8)	65 (10.9)	40 (58.0)	< 0.001
FFP ≥ 10 units, *n* (%)	126 (18.9)	96 (16.1)	30 (43.5)	< 0.001
PC ≥ 10 units, *n* (%)	69 (10.4)	48 (8.0)	21 (30.4)	< 0.001
TXA within 3 h, *n* (%)	232 (34.8)	195 (32.6)	37 (53.6)	0.001
Emergency procedures				
Craniotomy, *n* (%)	99 (14.9)	75 (12.6)	24 (34.8)	< 0.001
Thoracotomy, *n* (%)	17 (2.6)	5 (0.8)	12 (12.1)	< 0.001
Laparotomy, *n* (%)	35 (5.3)	25 (4.2)	10 (14.5)	< 0.001
Interventional radiology, *n* (%)	115 (17.3)	99 (16.6)	16 (23.1)	0.17

Data are expressed as numbers (%). Non-survivors are defined as patients who died within 28 days of injury. *FFP* Fresh frozen plasma, *PC* Platelet concentrate, *PRBCs* Packed red blood cells, *TXA* Tranexamic acid

### Univariate ROC curve analysis for 28-day mortality

**Fig. 2:**
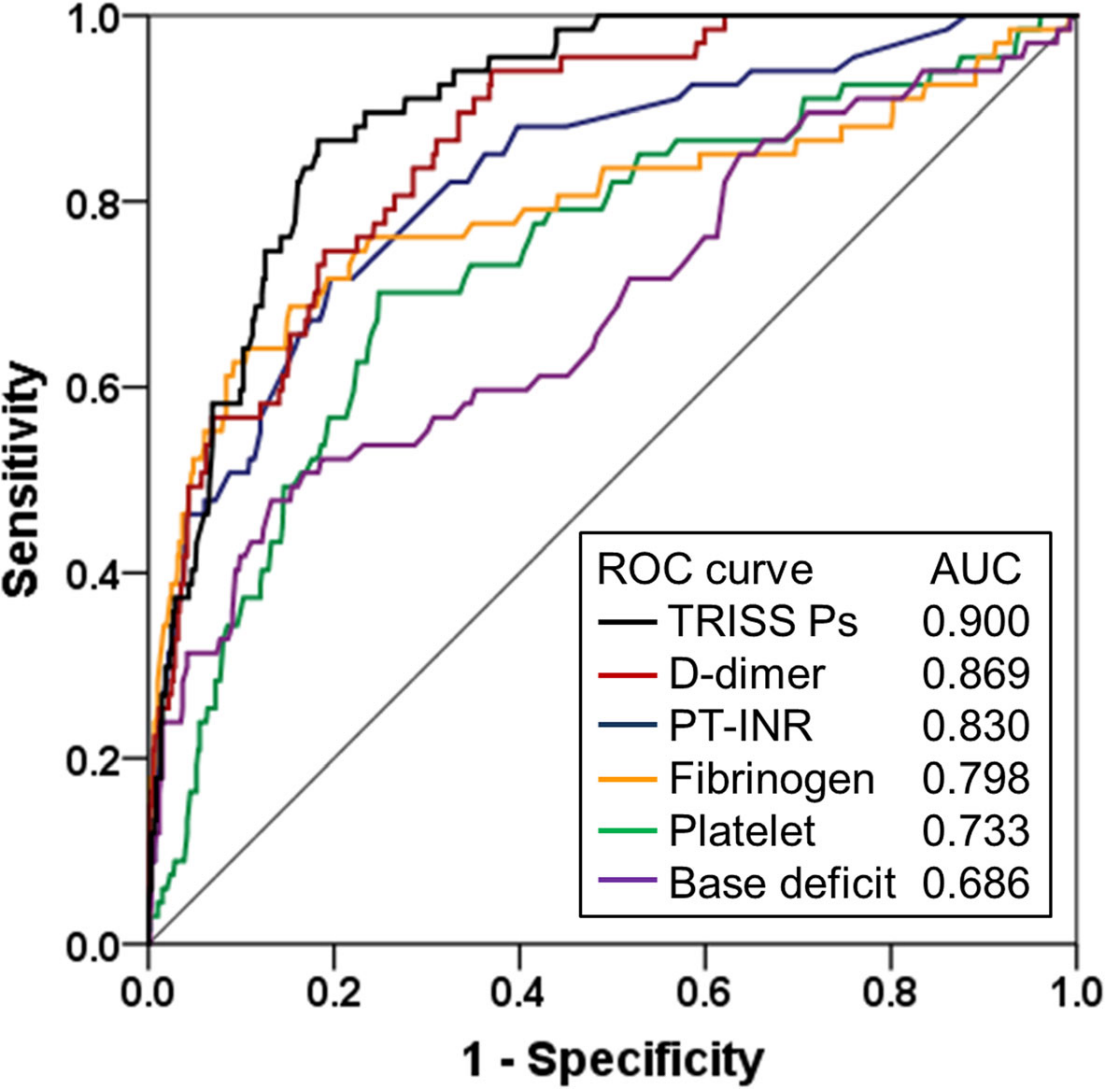
Univariate receiver operating characteristic (ROC) curve analysis for 28-day mortality. *AUC* area under the curve, *PT-INR* prothrombin time-international normalised ratio, *ROC* receiver operating characteristic, *TRISS Ps* probability of survival by trauma and injury severity score

The accuracy of variables, including coagulation and fibrinolytic markers for 28-day mortality, was evaluated using ROC curve analysis (Fig. 2). AUCs for platelet counts, fibrinogen, PT-INR and D-dimer were 0.733 (95% CI, 0.669–0.798), 0.798 (95% CI, 0.726–0.869), 0.830 (95% CI, 0.776–0.884) and 0.869 (95% CI, 0.829–0.909), respectively. Compared with base deficit (AUC, 0.686; 95% CI, 0.611–0.761), the AUCs were higher for fibrinogen (*p* = 0.006), PT-INR (*p* < 0.001) and D-dimer (*p* < 0.001).

### CART analysis for 28-day mortality

**Fig. 3:**
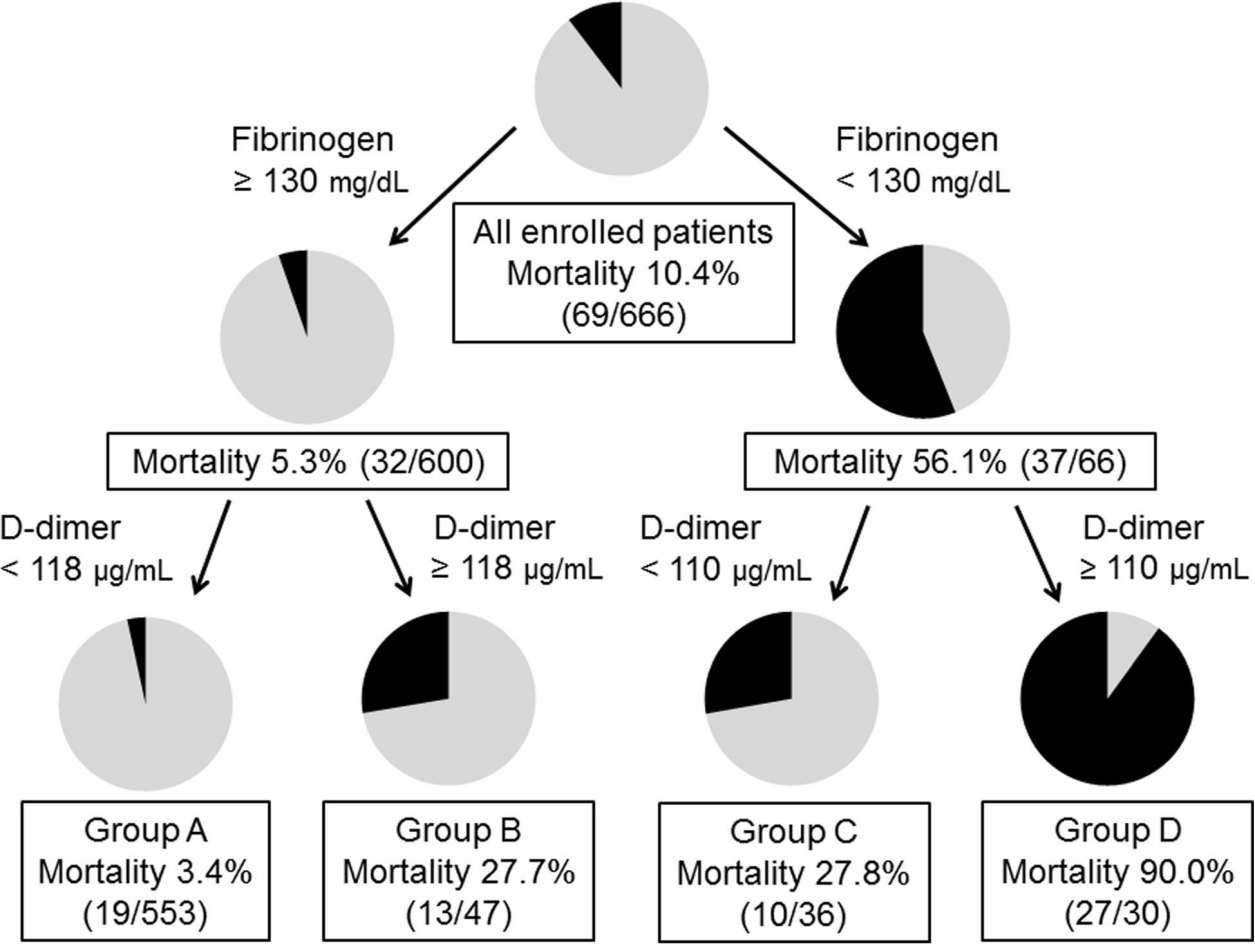
Classification and regression tree (CART) analysis for 28-day mortality. In the CART analysis, platelet counts, fibrinogen, prothrombin time-international normalised ratio, and D-dimer were injected as explanatory variables. The black portion in each pie chart represents 28-day mortality. The names of each terminal node are groups A, B, C and D. Group A is considered a low-risk group, and the groups B and C are moderate-risk groups and group D is considered a high-risk group

CART analysis revealed which sequences of coagulation and fibrinolytic markers best partitioned 28-day mortality risk in the format of decision trees (Fig. 3). The initial discriminator was fibrinogen of 130 mg/dL. D-dimer was identified as the second discriminators in both subgroups, and the cut-off values were 110 μg/mL in the lower fibrinogen subgroup (*n* = 66) and 118 μg/mL in the higher fibrinogen subgroup (*n* = 600). Finally, four terminal nodes were generated, and each terminal node was designated as follows: group A (higher fibrinogen/lower D-dimer, *n* = 553), group B (higher fibrinogen/higher D-dimer, *n* = 47), group C (lower fibrinogen/lower D-dimer, *n* = 36) and group D (lower fibrinogen/higher D-dimer, *n* = 30). The accuracy of the decision tree was 93.2%, and the 20-fold cross-validation estimated the misclassification cost (± standard error) at 6.9 ± 1.0%.

Table 3 presents the characteristics of the four groups. The 28-day mortality rates of groups A to D were low (19/553; 3.4%), moderate (13/47; 27.7%), moderate (10/36; 27.8%) and high (27/30; 90.0%), respectively. Despite including many multiple trauma patients in both groups, the major cause of death in group B differed from that in group C; all deaths in group B (13/13) were caused by TBI, whereas most deaths in group C (7/10) occurred because of exsanguination. Furthermore, significantly higher numbers of patients in groups B, C and D underwent massive transfusion compared with patients in group A (group A, 41 [7.1%] versus group B, 25 [53.2%], *p* < 0.001; group A versus group C, 21 [58.3%], *p* < 0.001; and group A versus group D, 17 [56.7%], *p* < 0.001).

**Table 3:** Characteristics of the four groups stratified using classification and regression tree analysis

	Group A *n* = 553	Group B *n* = 47	Group C *n* = 36	Group D *n* = 30
Injury location (AIS ≥ 3)				
Head, *n* (%)	367 (66.4)	35 (74.5)	20 (55.6)	28 (93.3)
Chest, *n* (%)	238 (43.0)	27 (57.4)	23 (63.9)	21 (70.0)
Abdomen, *n* (%)	70 (12.7)	14 (29.8)	14 (38.9)	7 (23.3)
Extremity/Pelvis, *n* (%)	134 (24.2)	23 (48.9)	21 (58.3)	6 (20.0)
Isolated TBI, *n* (%)	206 (37.3)	9 (19.1)	5 (13.9)	7 (23.3)
Multiple trauma, *n* (%)	240 (43.4)	35 (74.5)	26 (72.2)	23 (76.7)
Injury Severity Score	24 (17–29)	33 (26–43)	38 (25–56)	42 (31–53)
Blood transfusions within 24 h				
PRBCs ≥10 units, *n* (%)	41 (7.4)	25 (53.2)	21 (58.3)	17 (56.7)
FFP ≥ 10 units, *n* (%)	60 (10.8)	31 (66.0)	19 (52.8)	16 (53.3)
PC ≥ 10 units, *n* (%)	28 (5.1)	22 (46.8)	11 (30.6)	8 (26.7)
TXA within 3 h, *n* (%)	169 (30.6)	25 (53.2)	19 (52.8)	19 (63.3)
Mortality				
24-h mortality, *n* (%)	5 (0.9)	6 (12.8)	8 (22.2)	15 (50.0)
28-day mortality, *n* (%)	19 (3.4)	13 (27.7)	10 (27.8)	27 (90.0)
Cause of death				
Exsanguination, *n* (%)	2 (0.4)	0 (0)	7 (19.4)	2 (6.7)
TBI, *n* (%)	13 (2.4)	13 (27.7)	2 (5.6)	24 (80.0)
Sepsis/MODS, *n* (%)	3 (0.5)	0 (0)	0 (0)	1 (3.3)
Others, *n* (%)	1 (0.2)	0 (0)	1 (2.8)	0 (0)

Data are expressed as medians (25–75 percentiles) or numbers (%). Isolated TBI is defined as no injuries with an AIS score ≥ 3, except for the head injury. Multiple trauma is defined as multiple injuries with an AIS score ≥ 3 in two or more regions. Group A is the higher fibrinogen (≥ 130 mg/dL) and lower D-dimer (< 118 μg/mL) subgroup.Group B is the higher fibrinogen (≥ 130 mg/dL) and higher D-dimer (≥ 118 μg/mL) subgroup. Group C is the lower fibrinogen (< 130 mg/dL) and lower D-dimer (< 110 μg/mL) subgroup. Group D is the lower fibrinogen (< 130 mg/dL) and higher D-dimer (≥ 110 μg/mL) subgroup. *AIS* abbreviated injury scale, *CART* classification and regression tree, *FFP* fresh frozen plasma, *MODS* multiple organ dysfunction syndrome, *PC* platelet concentrate, *PRBCs* packed red blood cells, *TBI* traumatic brain injury, *TXA* tranexamic acid

### Multivariate logistic regression analysis for 28-day mortality

**Fig. 4:**
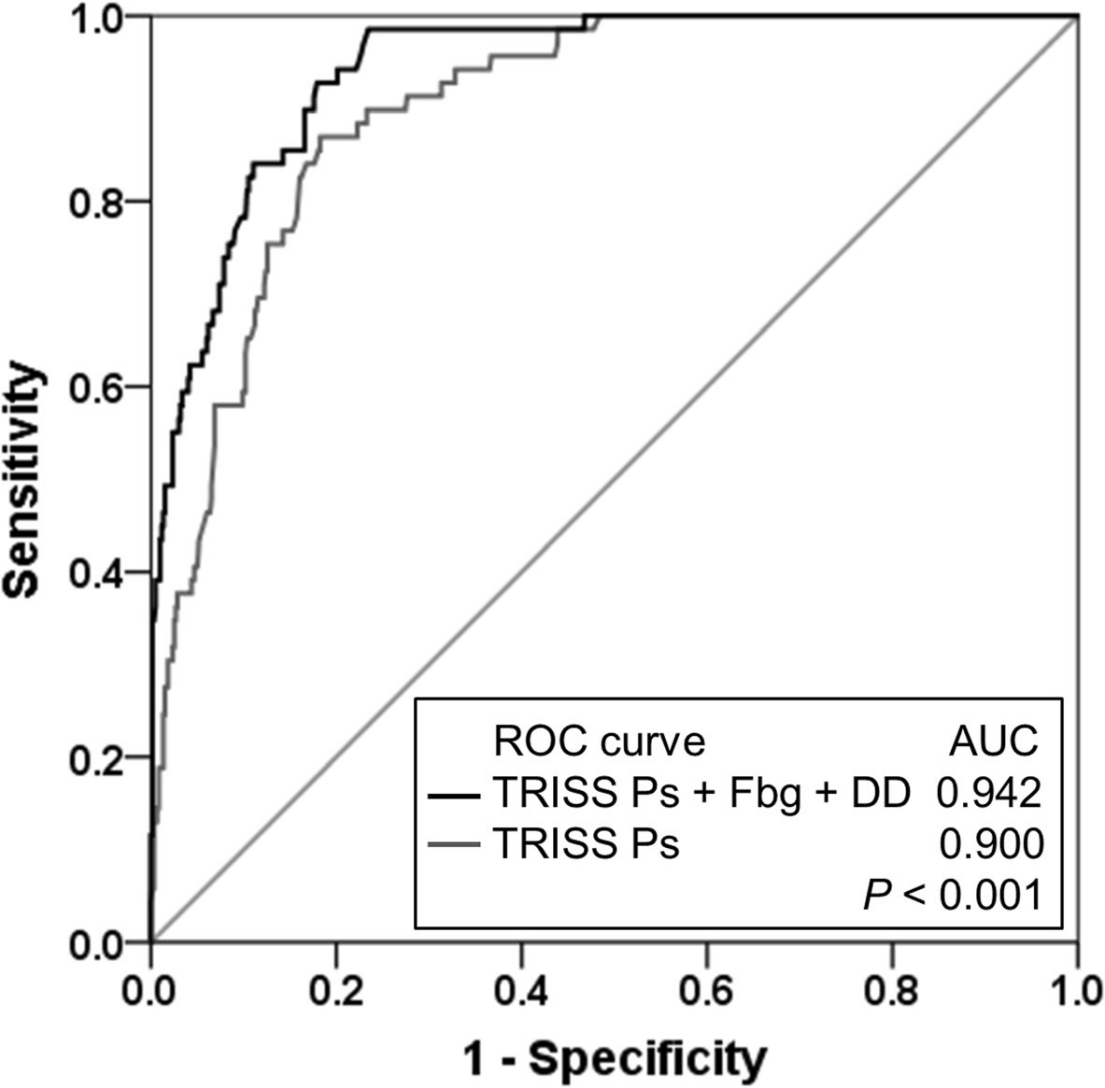
Receiver operating characteristic (ROC) curves of the prediction models for 28-day mortality. The multivariate logistic regression model (probability of survival calculated by trauma and injury severity score [TRISS Ps] plus fibrinogen and D-dimer) was compared with TRISS Ps alone. In the logistic regression model, fibrinogen and D-dimer were categorical variables and their cut-off values were 130 mg/dL and 110 μg/mL, respectively. *AUC* area under the curve, *DD* D-dimer, *Fbg* fibrinogen, *ROC* receiver operating characteristic, *TRISS Ps* probability of survival by trauma and injury severity score

The CART analysis identified two cut-off values of D-dimer (110 and 118 μg/mL), and we applied 110 μg/mL for further analysis. As revealed by the multivariate logistic regression analysis, low fibrinogen (< 130 mg/dL) and high D-dimer (≥ 110 μg/mL) were independently associated with 28-day mortality even after adjustment for TRISS Ps (Table 4). This logistic regression model demonstrated a significantly higher AUC (0.942, 95% CI, 0.920–0.964) for the ROC analysis than the TRISS Ps alone (0.900, 95% CI, 0.870–0.931; *p* < 0.001; Fig. 4). The addition of fibrinogen and D-dimer to the TRISS Ps yielded a significant NRI (1.215, 95% CI, 0.998–1.432; *p* < 0.001) and IDI (0.180, 95% CI, 0.113–0.247; *p* < 0.001).

**Table 4:** Multivariate logistic regression analysis on 28-day mortality with adjustment for TRISS Ps

	Co-efficient (β)	Adjusted OR	95% CI	*P* value
TRISS Ps	−3.86	0.02	0.007–0.05	< 0.001
Fibrinogen < 130 mg/dL	2.26	9.55	4.50–22.60	< 0.001
D-dimer > 110 μg/mL	1.77	5.89	2.78–12.70	< 0.001
Constant	−0.84			0.04

*CI* Confidence interval, *OR* Odds ratio, *TRISS Ps* Probability of survival by trauma and injury severity score

## Discussion

In the present study, we investigated the impact of initial coagulation and fibrinolytic markers including platelet counts, PT-INR, fibrinogen and D-dimer on 28-day mortality in patients with severe blunt trauma. CART analysis revealed that fibrinogen and D-dimer are the most profitable discriminators among these markers for predicting 28-day mortality. Although there were significant difference in platelet count and PT-INR between survivors and non-survivors, CART analysis did not identified these two markers suggesting that the combination of fibrinogen and D-dimer predicted 28-day mortality the best. Furthermore, low fibrinogen and high D-dimer were the useful for predicting mortality even after adjustment of the patient severity calculated by the TRISS Ps.

Fibrinogen is essential for clot formation and platelet aggregation; during major blood loss, fibrinogen decreases to critically low levels earlier than the other coagulation factors [25] and the early phase of severe trauma [26]. Although several studies report the benefits of fibrinogen concentrate or cryoprecipitate transfused in patients with hypofibrinogenaemia, because these productions contain abundant fibrinogen [8, 27, 28], the optimal threshold for treating fibrinogen depletion remains variable and opinion-based. The 2015 guidelines from the American Society of Anaesthesiologists suggested a threshold of 80–100 mg/dL [29], whereas the 2016 European guidelines recommended initiating fibrinogen supplementation when fibrinogen level is < 150–200 mg/dL [18]. Our results proposed a cut-off value of 130 mg/dL; the 28-day mortality was 5.3% in patients with initial fibrinogen levels ≥130 mg/dL, whereas 56.1% of the patients died when initial fibrinogen levels were <  130 mg/dL. These results suggested that fibrinogen supplementation should be initiated at least before the plasma fibrinogen level decreases to a critical level of 100 mg/dL. Further studies are warranted to evaluate the validity of early fibrinogen supplementation, including fibrinogen concentrates and cryoprecipitate activated by initial fibrinogen levels of 100–150 mg/dL.

The second discriminator in the CART analysis was the D-dimer. In the subgroup analysis, we found that all patients with high a D-dimer without fibrinogen depletion died of TBI. Because the D-dimer is generated as a result of fibrin formation and sequential fibrinolysis [30], it is considered a useful marker for excessive fibrinolysis in patients with trauma [12]. Thus, hyperfibrinolysis should be corrected in patients with high D-dimer to prevent death due to TBI. An important treatment option in such a situation is an antifibrinolytic drug, including tranexamic acid [31]. Although recent trials have failed to demonstrate the efficacy of tranexamic acid on clinical outcomes in patients with TBI [32, 33], it is possible that selective use of the drug in the TBI patients with hyperfibrinolysis provides benefits. Our results suggested that patients with high D-dimer levels and TBI are potential candidates for TXA administration, because they have evidence of hyperfibrinolysis and are at high risk for mortality from TBI. Thus, the efficacy of selective use of TXA should be investigated in future studies.

The association between the types of coagulation and fibrinolytic abnormalities and cause of death in trauma patients has not been extensively investigated. We found that patients with sustained fibrinogen and high D-dimer died due to TBI, whereas majority of patients with decreased fibrinogen without elevated D-dimer died from exsanguination. The coagulation system is usually activated in patients with severe trauma, converting fibrinogen to fibrin to form blood clots [34]. TBI predisposes patients to hyperfibrinolysis following activation of the coagulation system, known as secondary hyperfibrinolysis, that degrades fibrin to D-dimer [13]. Severe haemorrhagic shock causes tissue hypoxia/ischemia injury that leads to increased release of tissue-plasminogen activator from endothelial cells [35]. This excessive fibrinolysis breaks down not only fibrin but also fibrinogen. We speculate that patients with sustained fibrinogen and elevated D-dimer had secondary hyperfibrinolysis that worsened the TBI and that patients with decreased fibrinogen without elevated D-dimer had primary hyperfibrinolysis caused by haemorrhagic shock that resulted in death from exsanguination.

Our study includes several limitations that might cause a bias in the results or interpretations. First, this was a retrospective cohort study conducted at only two tertiary care hospitals. In particular, optimal cut-off values of these markers can differ according to patient characteristics and background of each study cohort. External validation should be performed to confirm the results. Second, in the two hospitals, the D-dimer level was quantified using different reagents at the two companies. The difference may affect the results in this study; however, we could not adjust the difference because there is no established method. Third, we could not investigate effects of anticoagulant/platelet agents on mortality as small number of patients used these drugs before injury. Against our expectations, the proportion of patients who used these drugs was slightly lower in non-survivors than survivors; however, the association of antithrombotic agents with outcomes in patients should be explored separately. Fourth, we examined only the initial coagulation and fibrinolytic markers measured upon ED arrival and did not routinely examine other important markers, such as fibrinogen and fibrin degradation products (FDP), thrombin–antithrombin complex and plasminogen activation inhibitor. As FDP reflects primary hyperfibrinolysis as well as secondary hyperfibrinolysis, it may be a better predictor than D-dimer. The impact of FDP on mortality should also be investigated. Finally, only patients with severe blunt trauma were included. As a result, most patients in this study cohort had TBI. It was thus not possible to conduct a subgroup analysis to evaluate the impact of the tested markers on death from exsanguination in patients without TBI. Further studies are warranted to examine whether our findings are applicable to severe haemorrhagic shock patient population including many penetrating injuries and/or fewer TBI.

## Conclusions

Fibrinogen and D-dimer were the principal markers for stratification of the risk of death in patients with severe blunt trauma. Even after adjustment for TRISS Ps, fibrinogen depletion and D-dimer elevation were independently associated with mortality; they might play critical roles in haemostatic abnormalities in the early phase of trauma and could be the therapeutic targets and predictive variables of mortality.

## Abbreviations

AIS: Abbreviated injury scale
AUC: Area under the curve
CART: Classification and regression tree
CI: Confidence interval
ED: Emergency department
FFP: Fresh frozen plasma
IDI: Integrated discrimination improvement
IQR: Interquartile range
ISS: Injury severity score
MODS: Multiple organ dysfunction syndrome
NRI: Net reclassification improvement
OR: Odds ratio
PC: Platelet concentrate
PT-INR: Prothrombin time-international normalised ratio
ROC: Receiver operating characteristic
TBI: Traumatic brain injury
TRISS Ps: Probability of survival by the trauma and injury severity score
